# tealeaves: an R package for modelling leaf temperature using energy budgets

**DOI:** 10.1093/aobpla/plz054

**Published:** 2019-12-08

**Authors:** Christopher D Muir

**Affiliations:** Department of Botany, University of Hawai’i, Honolulu, HI, USA

**Keywords:** Boundary layer, energy balance, leaf size, leaf temperature, mathematical model, plant leaves, plant physiology, R

## Abstract

Plants must regulate leaf temperature to optimize photosynthesis, control water loss and prevent damage caused by overheating or freezing. Physical models of leaf energy budgets calculate the energy fluxes and leaf temperatures for a given set leaf and environmental parameters. These models can provide deep insight into the variation in leaf form and function, but there are few computational tools available to use these models. Here I introduce a new R package called **tealeaves** to make complex leaf energy budget models accessible to a broader array of plant scientists. This package enables novice users to start modelling leaf energy budgets quickly while allowing experts to customize their parameter settings. The code is open source, freely available and readily integrates with other R tools for scientific computing. This paper describes the current functionality of **tealeaves**, but new features will be added in future releases. This software tool will advance new research on leaf thermal physiology to advance our understanding of basic and applied plant science.

## Teaser

**tealeaves** is a new R package to implement complex, customizable leaf energy budget models as part of an open source, transparent workflow.

## Introduction

Plants grow, survive and reproduce under a wide variety of temperatures because natural selection endows them with adaptations to cope with different thermal regimes. Cushion plants in the alpine grow near the ground to stay warm, desert plants decrease absorptance to stay cool (Ehleringer *et al.* 1976) and plants keep stomata open, which can protect against extreme heat waves (Drake *et al.* 2018). Understanding these diverse mechanisms of thermal adaptation and acclimation may provide insight into how plants respond to increasing temperatures and how these responses influence ecosystem function with anthropogenic climate change (Rogers *et al.* 2017; Crous 2019). Because leaves are the primary photosynthetic organ in most plants, regulating leaf temperature is critical (Berry and Björkman 1980). Photosynthesis peaks at intermediate temperatures (Sage and Kubien 2007). When leaves are too warm, evaporation increases exponentially, photo- and non-photorespiratory losses subtract from carbon gain (Jones 2014) and critical loss of function occurs about ~50 °C (O’Sullivan *et al.* 2017). When leaves are too cold, maximum photosynthetic rates decline and can lead to damage from excess solar radiation (Huner *et al.* 1993) as well as nighttime dew and frost formation (Jordan and Smith 1994). Natural selection should favor leaf morphologies and physiological responses that optimize leaf temperature in a given environment (Parkhurst and Loucks 1972; Okajima *et al.* 2012; Michaletz *et al.* 2016).

To understand leaf thermal physiology, plant scientists need mathematical and computational tools to model leaf temperature as a function of leaf traits and the environment. Balancing energy budgets is a powerful mathematical tool for understanding how leaf traits and environmental parameters influnce plant physiology that has been used for over a century (Raschke 1960). The equilibrium leaf temperature is that in which the energy gained from incoming solar and infrared radiation is balanced by thermal infrared radiation losses, sensible heat loss/gain and latent heat loss through evaporation (Gutschick 2016). Leaf angle, size and conductance to water vapour alter leaf temperature by changing how much solar radiation they intercept and how much heat they lose through sensible and latent heat flux. Likewise, enrvironmental factors such as sunlight, air temperature, humidity and wind speed influence heat transfer between leaves and the surrounding microclimate (Gutschick 2016). Under controlled conditions, leaf energy budget models are highly accurate (Schymanski and Or 2017). Hence, they can offer deep insight on plant thermal physiology by asking how temperature is affected by one factor in isolation or in combination with another.

Leaf energy budget models have many applications, but perhaps their most widespread use is in modelling optimal leaf size and shape. The boundary layer of still air just above and below the leaf surface determines sensible and latent heat transfer and is proportional to leaf size (Gates 1968). All else being equal, larger leaves have a thicker boundary layer, slowing heat transfer and decoupling leaf temperature from air temperature. This likely explains why, for example, many warm desert species have small leaves (Gibson 1998). Using leaf energy budgets, Parkhurst and Loucks (1972) further predicted that leaves should be small in cold air and large under warm, shaded conditions. More recently, Okajima *et al.* (2012) extended these models, showing that small leaves maximize photosynthetic rate under high insolation and warm temperatures, but large leaves increase water-use efficiency in shadier habitats. Wright *et al.* (2017) used energy budget models to show that dew and frost formation may select against large leaves at high latitudes. Energy budget models also help explain variation in leaf shape, such as lobing and dissection, because heat transfer is determined by effective leaf width (aka characteristic leaf dimension; Taylor 1975) rather than total area. Effective leaf width is ‘the diameter of the largest circle that can be inscribed within the margin’ (Leigh *et al.* 2017). Lower effective leaf width reduces leaf temperature under natural conditions in the sun (Leigh *et al.* 2017) and is under selection in sunny, drier habitats (Ferris *et al.* 2015). Besides leaf size and shape, energy balance models are useful in understanding many plant processes and traits (Gates 1965), such as transpiration (Gates 1968), optimal stomatal conductance (Buckley *et al.* 2014), stomatal arrangements (Foster and Smith 1986), leaf thickness (Leigh *et al.* 2012), response to sunflecks (Schymanski *et al.* 2013), carbon economics (Michaletz *et al.* 2016) and water-use efficiency (Schymanski and Or 2016).

Despite the utility of leaf energy budget models, there are a dearth of open source, customizable, computational tools to implement them. The **plantecophys** package implements a similar energy budget model (Duursma 2015). However, the model is simplified for faster computation needed in ecosystem and global land surface models (Leuning *et al.* 1995). Therefore, it does not incorporate features such as different boundary layer conductances on each leaf surface, nor can users easily change default parameters for specialized cases. The Landflux website also has an Excel spreadsheet for leaf energy budgets (Tu 2019), but it is prohibitively time-consuming and not reproducible to use spreadsheets for large-scale simulations. Because computational tools are limited, potential users must develop models anew and learn the numerical methods necessary to find solutions. Ideally, there should be a platform in which novices can model leaf temperature to solve an interesting problem without having to write their own model and learn complicated numerical algorithms. At the same time, we need a platform that can be easily modified for experts that want to extend existing leaf energy balance models.

The goal of this paper is therefore to develop software that models leaf temperature as a function of leaf traits and the environment with physical realism. This software should be open source so that the methods are transparent and code can be modified by other researchers. Secondly, it should be readily available to novice modelers yet customizable by those working on more specific problems. Finally, it should easily integrate with other advanced tools for scientific computing. To that end, I developed an R package called **tealeaves** to model leaf temepature in response to a wide variety of leaf and environmental parameters. The source code is open source and available to modify; it is easy to use with default parameters, but also customizable; and because it is written in R, the output from **tealeaves** can be analysed and visualized with the vast array of computational tools availble in the R environment.

## Methods

Annotated source code to generate this manuscript is available on GitHub (https://github.com/cdmuir/tealeaves-ms).

**Figure 1.**
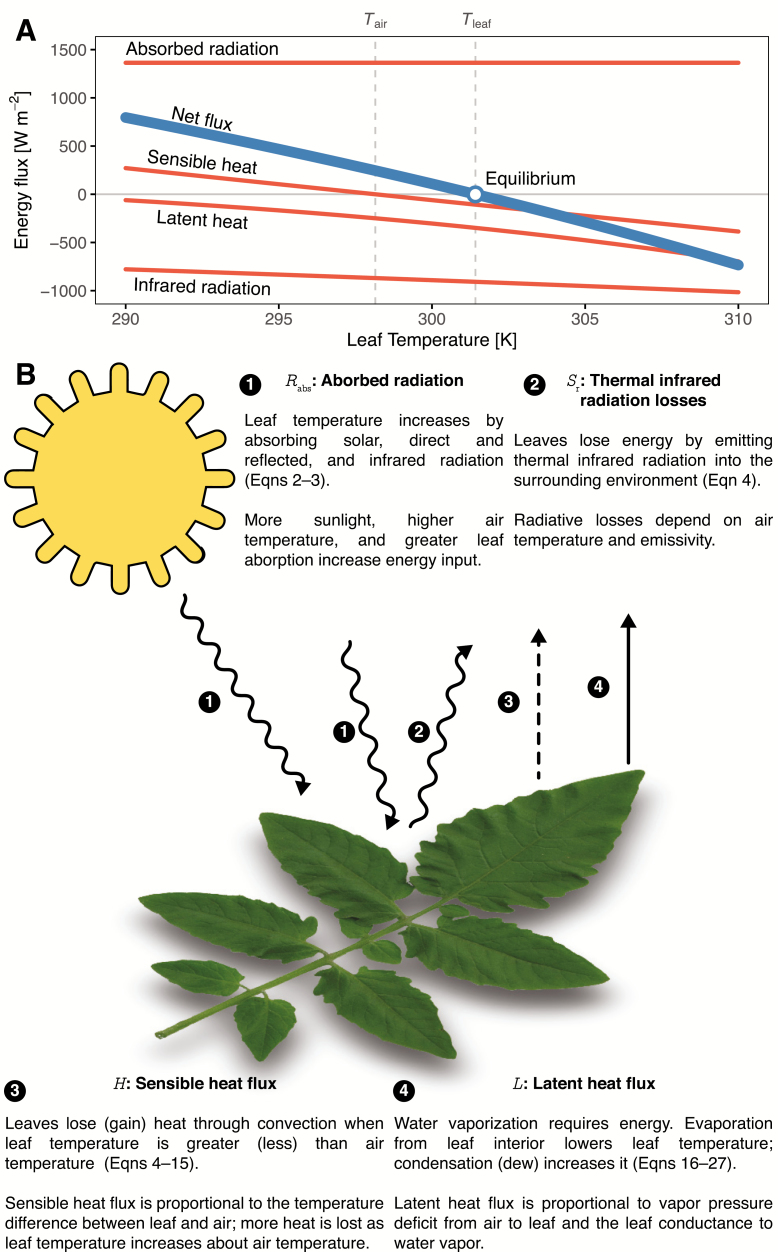
The leaf energy budget model in **tealeaves** takes environmental and leaf parameters to find the equilibrium leaf temperature ($T_{leaf}$) at which incoming energy exactly matches outgoing energy. Under typical daytime conditions, this means thermal infrared radiation losses, sensible heat loss and latent heat loss must balance absorbed radiation. (A) The components of leaf energy budgets (thin orange lines) vary with leaf temperature (*x*-axis). $T_{leaf}$ is where the net energy flux (thick blue line) is 0 (blue point; see Equation 1). (B) 1) Radiation is emitted from sunlight and surrounding objects is absorbed by the leaf. 2) The leaf radiates some energy. 3) Heat is lost through convection when the leaf temperature is greater than air temperature. 4) Latent heat is lost through evaporation, which is driven by the vapor-pressure deficit between the air and leaf interior and the leaf conductance to water vapor. Calculations used the following leaf parameter values in this example: *d* = 0.1 m; $\alpha_{s}=0.5$; $\alpha_{l}=0.97$; $g_{sw}=5\;\mu mol\;m^{-2}\;s^{-1}\;Pa^{-1}$; $g_{uw}=0.1\;\mu mol\;m^{-2}\;s^{-1}\;Pa^{-1}$; SR=0.5. Calculations used the following environmental parameter values in this example: *P* = 101.3246 kPa; *r* = 0.2; RH=0.5; $S_{sw}=1000$ W m^−2^; $T_{air}=298.15$ K; *u* = 2 m s^−1^. See Table 1 for symbol definitions.

Leaf energy budgets consist of incoming and outgoing energy fluxes. Incoming energy includes radiation from solar (aka short-wave) and thermal infrared (aka long-wave) sources. Outgoing energy includes losses of infrared radiation, sensible heat and latent heat flux due to evaporation (Fig. 1). Note that sensible and latent fluxes can be positive when leaves are warmer than the air or condensation (dew) occures, respectively. When leaves reach a thermal equilibrium with their environment—generally within a few minutes—these incoming and outgoing energy sources balance one another. Formally, one solves for the leaf temperature ($T_{leaf}$) that balances the energy budget:

**Table 1.** Parameter inputs for **tealeaves**. Each parameter has a mathematical symbol used in the text, the R character string used in the **tealeaves** package, a brief description and the units. For physical constants, a value is provided where applicable, though users can modify these if desired.

Symbol	R character	Description	Units
Leaf parameters:			
*d*	leafsize	Leaf characteristic dimension	m
$\alpha_{l}$	abs_l	Absorbtivity of long-wave radiation (4–80 µm)	None
$\alpha_{s}$	abs_s	Absorbtivity of short-wave radiation (0.3–4 µm)	None
$g_{sw}$	g_sw	Stomatal conductance to water vapour	µmol m^−2^ s^−1^ Pa^−1†^
$g_{uw}$	g_uw	Cuticular conductance to water vapour	µmol m^−2^ s^−1^ Pa^−1†^
SR		Stomatal ratio (untransformed)	None
sr	logit_sr	Stomatal ratio (logit transformed)	None
Environmental parameters:			
*P*	P	Atmospheric pressure	kPa
*r*	r	Reflectance for short-wave irradiance (albedo)	None
RH	RH	Relative humidity	None
*S*_sw_	S_sw	Incident short-wave (solar) radiation flux density	W m^−2^
*T*_air_	T_air	Air temperature	K
*u*	wind	Wind speed	m s^−1^
Physical constants:			
*a*, *b*, *c*, *d*	a, b, c, d	Coefficients for calculating Nu and Sh numbers	None
*c*_*p*_	c_p	Heat capacity of air	1.01 J g^−1^ K^−1^
*D*_h,0_	D_h0	Diffusion coefficient for heat in air at 0 °C	19.0 × 10^−6^ m^2^ s^−1^
*D*_m,0_	D_m0	Diffusion coefficient for momentum in air at 0 °C	13.3 × 10^−6^ m^2^ s^−1^
*D*_w,0_	D_w0	Diffusion coefficient for water vapour in air at 0 °C	21.2 × 10^−6^ m^2^ s^−1^
ε	epsilon	Ratio of water to air molar masses	0.622
*eT*	eT	Exponent for temperature dependence of diffusion	1.75
*G*	G	Gravitational acceleration	9.8 m s^−2^
$\bar{R}$	R	Ideal gas constant	8.3144598 J mol^−1^ K^−1^
*R*_air_	R_air	Specific gas constant for dry air	287.058 J kg^−1^ K^−1^
*σ*	s	Stefan–Boltzmann constant	5.67 × 10^−8^ W m^−2^ K^−4^

^†^Conductances are presented in molar units for consistency with literature on photosynthesis but are converted to m s^−1^ using the ideal gas law (see text for details) to match conductance to heat transfer.

$$\begin{array}{l} 0=R_{abs}-(S_{r}+H+L). \end{array}$$ (1)

$R_{abs}$ is the absorbed radiation, $S_{r}$ is thermal infrared radiation loss, *H* is sensible heat flux and *L* is latent heat flux. All of these values have units W m^−2^. Environmental and leaf parameters like irradiance, air temperature and leaf absorbtivity determine how much energy is absorbed and radiated. In the daytime, radiation coming into the leaf generally exceeds that coming out, resulting in a leaf temperature above air temperature. Sensible heat loss (gain) occurs when the leaf is warmer (cooler) than the surrounding air, but the rate is influenced by other parameters like wind speed and leaf size. Latent heat loss occurs when the water vapor pressure of the surrounding air is lower than that in the leaf, driving evaporation. Relative humidity of the air and leaf conductance to water vapour determine how strong the vapor-pressure deficit is and how much resistance to water transport occurs. Leaves gain energy when water condenses as dew. The leaf energy balance model works by taking a set of environmental and leaf parameters, then finding the leaf temperature at which they balance one another. The equilibrium leaf temperature can be well above air temperature under high irradiance, low wind speed and/or low conductance to water vapor; leaf temperature can be near or even below air temperature under low irradiance, in small leaves and/or leaves with high rates of evaporation.

The primary aim of this paper is not to extend leaf energy budget theory, but to describe the **tealeaves** package which implements existing models. Therefore, the mathematical details behind the model are provided in an Appendix at the end of this paper. Tables 1 and 2 list all mathematical symbols in parameter inputs and calculated output values. **Supporting Information—Table S1** lists current default parameter values and realistic ranges with references to the literature. These equations describe the current **tealeaves** implementation. As future releases will alter some assumptions and incorporate new features, I mention future modifications in the Discussion. In this section, I describe how leaf energy budget models are implemented in R and provide worked examples.

**Table 2.** Calculated parameters and outputs for **tealeaves**. Some parameters are intermediate calculations (see Methods) but are not included in the **tealeaves** output (see R documentation accompanying package for further detail). Each parameter has a mathematical symbol used in the text, the R character string used in the **tealeaves** package, a brief description and the units.

Symbol	R character	Description	Units
Leaf parameters:			
*E*	E	Transpiration rate	mol m^−2^ s^−1^
*g*_h_	g_h	Boundary layer conductance to heat	m s^−1^
*g*_bw_	g_bw	Boundary layer conductance to water vapour	m s^−1^
*g*_tw_	g_tw	Total conductance to water vapour	m s^−1^
Gr	Gr	Grashof number	None
*H*	H	Sensible heat flux	W m^−2^
*L*	L	Latend heat flux	W m^−2^
Nu	Nu	Nusselt number	None
*R*_abs_	R_abs	Absorbed radiation	W m^−2^
Re	Re	Reynolds number	None
*S*_r_	S_r	Thermal infrared radiation losses	W m^−2^
Sh	Sh	Sherwood number	None
*T*_leaf_	T_leaf	Leaf temperature	K
Environmental parameters:			
*d*_wv_	d_wv	Water vapour pressure differential	mol m^−3^
*h*_vap_	h_vap	Latent heat of vapourization	J mol^−1^
*P*_a_	P_a	Density of dry air	g m^−3^
*p*_air_	p_air	Water vapour pressure of the air	kPa
*p*_sat_	p_sat	Saturating water vapour pressure	kPa
*S*_lw_	S_lw	Incident long-wave (thermal infrared) radiation flux density	W m^−2^
*T*_sky_	T_sky	Clear sky temperature	K
Physical constants:			
*D*_h_	D_h	Diffusion coefficient for heat in air at a given temperature	m^2^ s^−1^
*D*_m_	D_m	Diffusion coefficient for momentum in air at a given temperature	m^2^ s^−1^
*D*_w_	D_w	Diffusion coefficient for water vapour in air at a given temperature	m^2^ s^−1^
Convergence diagnostics:			
	value	Energy balance at equilibrium *T*_leaf_	W m^−2^
	convergence	0 = converged; 1 = failed	none

### Solving in R

R is a fully open source programming language for statistical computing that allows users to develop their own packages with new functions. **tealeaves** takes three sets of parameter inputs: leaf parameters, environmental parameters and physical constants (see Table 1). The package provides reasonable defaults, but users can input new values to address their question, as I demonstrate in the next section. With one or more parameter sets, **tealeaves** uses the uniroot function in R base package **stats** to find the $T_{leaf}$ that balances the leaf energy budget (Equation 1). It outputs the equilibrium $T_{leaf}$ and energy fluxes in a table for analysis and visualization.

Unlike previous leaf energy models, **tealeaves** ensures that calculations are technically correct by assigning stadard SI units with the R package **units**Pebesma *et al.* (2016). Every parameter and calculated value must have correctly assigned units. If units are not properly defined, **tealeaves** will produce an error because it is unable to convert values. For speed, calculations can optionally be made without units and will yield correct results if provided values have correct units. To ensure accuracy, these unitless functions are tested against their counterparts with units using the **testthat** package (Wickham 2011). Other R packages that contributed to **tealeaves** are **crayon** (Csárdi 2017), **dplyr** (Wickham *et al.* 2019), **glue** (Hester 2019), **furrr** (Vaughan and Dancho 2018), **future** (Bengtsson 2019), **ggplot2** (Wickham 2016), **magrittr** (Bache and Wickham 2014), **purrr** (Henry and Wickham 2019a), **rlang** (Henry and Wickham 2019b), **stringr** (Wickham 2019), **tidyr** (Wickham and Henry 2019).

### Worked examples

In this section, I provide two worked examples; more complex worked examples are found in the **Supporting Information**. The first illustrates that it is straightforward to use **tealeaves** with a few lines of code with default settings. The second shows that it is also possible to model $T_{leaf}$ across multiple leaf trait and environmental gradients for more advanced applications.

#### Example 1: a minimum worked example.

The box below provides R code implementing the minimum worked example with default settings.


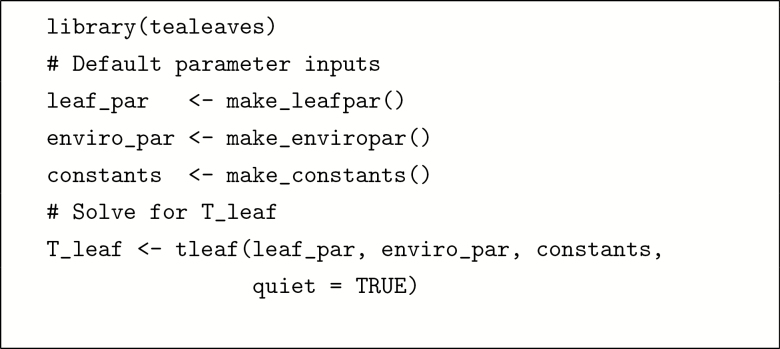


#### Example 2: leaf temperature along environmental gradients.

The box below provides R code to calculate leaf temperature along an air temperature gradient for leaves of different sizes.


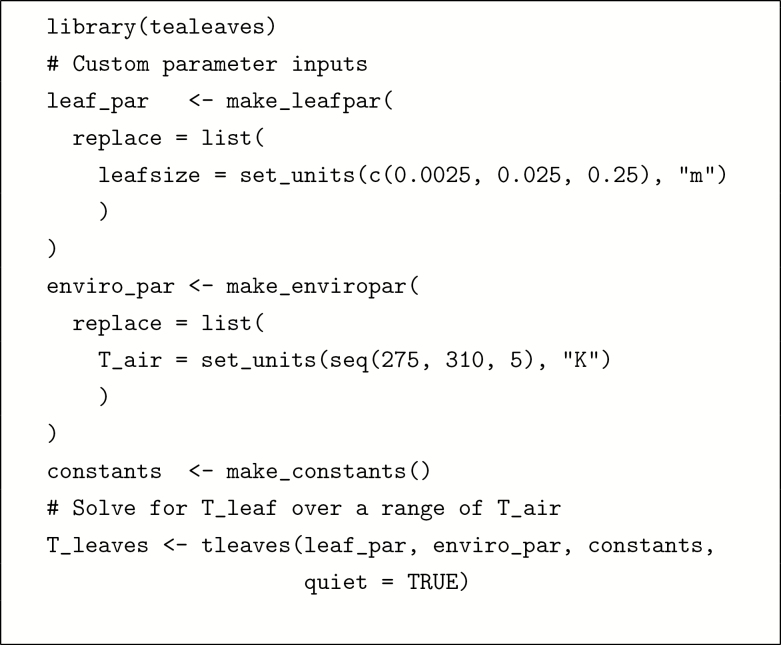


### Extended examples

To see the range of possible applications for **tealeaves**, I ran four additional sets of simulations. The first models the leaf-to-air temperature differential for different leaves sizes across a gradient of air tempuratures; the second models the leaf-to-air temperature differential across a gradient of incident solar radiation for different stomatal conductances; the third models the leaf-to-air temperature differential for different-sized leaves under free, mixed and forced convection; and the fourth models the effect of stomatal ratio on evaporation under free and forced convection. These extended examples are documented more fully in the **Supporting Information** with accompanying R code.

To provide a sense of which leaf and environmental parameters affect $T_{leaf}$ the most under ‘typical’ conditions, I varied stomatal conductance ($g_{sw}$), leaf size (*d*), stomatal ratio (SR), relative humidity (RH), solar radiation ($S_{sw}$) and wind speed (*u*) over a wide range of realistic values while holding all other values constant at their default setting **[see****Supporting Information—Table S1****]**.

## Results

### tealeaves’s source code is open to all

A development version of **tealeaves** is currently available on GitHub (https://github.com/cdmuir/tealeaves). A stable version of **tealeaves** will be released on the Comprehensive R Archive Network (CRAN, https://CRAN.R-project.org/package=tealeaves). I will continue developing the package and depositing revised source code on GitHub between stable release versions. Other plant scientists can contribute code to improve **tealeaves** or modify the source code on their own installations for a more fully customized implementation.

### tealeaves is straightforward to use and modify

**Figure 2.**
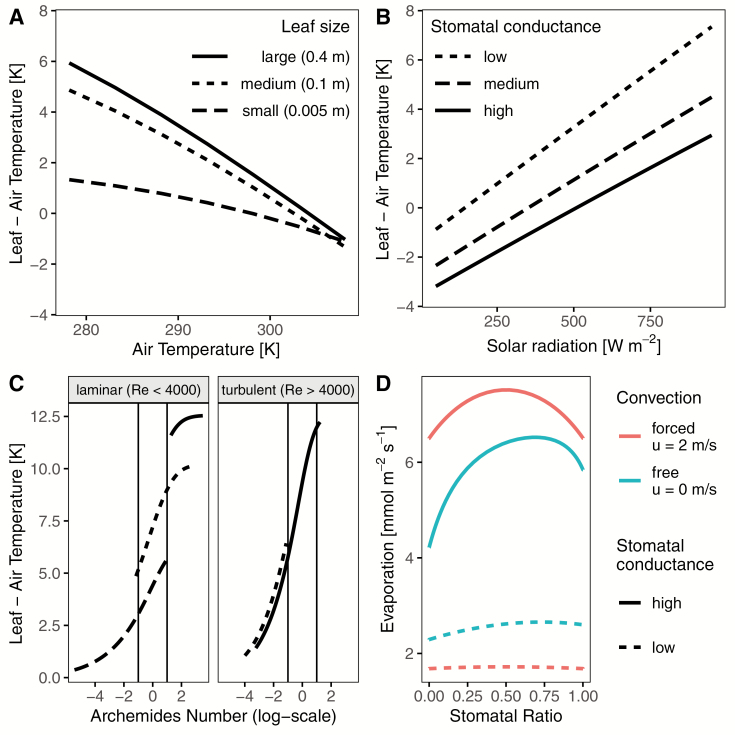
Extended examples of **tealeaves**. Code to generate these examples is provided in the **Supporting Information**. (A) The temperature of smaller leaves is more closely coupled to air temperature. Each line represents a different leaf size (small, dashed line; medium, dotted line; large, solid line) and the leaf-to-air temperature differential (*y*-axis) over an air temperature gradient (*x*-axis). (B) Greater stomatal conductance cools leaves. Each line represents a different stomatal conductance (low, dashed line, 1 µmol m^−2^ s^−1^ Pa^−1^; medium, dotted line, 3 µmol m^−2^ s^−1^ Pa^−1^; high, solid line, 5 µmol m^−2^ s^−1^ Pa^−1^) and the leaf-to-air temperature differential (*y*-axis) over a gradient of incident solar radiation (*x*-axis). (C) Forced convection dominates in small leaves; free convection dominates in very large leaves. Leaf size is indicated by line type as in Panel (A). Vertical lines indicate approximate shifts from forced convection (Ar<0.1), mixed convection (0.1<Ar<10) and free convection (Ar>10). Small leaves always experience forced convection, leading to lower leaf temperature compared to large leaves experiencing free convection. (D) Amphistomatous leaves (stomatal ratio ~ 0.5) evaporate more than hypo- or hyperstomatous leaves (stomatal ratio ~ 0 or 1, respectively), especially under free convection (low wind speed, *u*).

**tealeaves** lowers the activation energy to start using leaf energy budgets in a transparent and reproducible workflow. Default settings provide a reasonable starting point (see Worked Example 1 and **Supporting Information—Table S1**), but they should be carefully inspected to ensure that are appropriate for particular questions. At default settings, low stomatal conductance, high humidity and/or low wind speed cause leaf temperatures to heat substantially above air temperature **[see****Supporting Information—Fig. S1A, D and F****]**. Small leaves are closely coupled to air temperature, whereas large leaves are not **[see****Supporting Information—Fig. S1B****]**. Leaves can operate below air temperature at low light, but above it at higher light **[see****Supporting Information—Fig. S1E****]**. Stomatal ratio has only a modest effect on leaf temperature **[see****Supporting Information—Fig. S1C****]**. Most users will want to modify these default settings and simulate leaf temperature over a range of leaf and environmental parameters, so these results are not generalizable to all cases. By design, **tealeaves** easily allows users to define multiple simultaneous trait and environmental gradients (see Worked Example 2 and Fig. 2).

## Discussion

Scientists have used energy budgets to model leaf temperature for over a century (see Raschke (1960) for historical references). Despite many advances in our understanding of the environmental and leaf parameters that affect heat exchange (Gutschick 2016), there exist few computational tools to implement complex energy budget models. The **tealeaves** package fills this gap by providing a platform for modelling energy budgets in a transparent and reproducible way with R (R Core Team 2019), a freely available and widely used programming language for scientific computing. Unlike previous software, **tealeaves** removes ambiguity by forcing users to specify proper SI units through the R package **units** (Pebesma *et al.* 2016). Neophytes with little experience modelling leaf temperature may get started quickly without having to develop their model *de novo*, while specialists can modify the code to customize **tealeaves** to their specificiations. **tealeaves** also readily integrates with the vast array of data analysis and visualization tools in R. These features will enable wider adoption of leaf energy budgets models to understand plant biology. However, as I discuss below, the current version of **tealeaves** has several important limitiations that can be addressed in future releases.

Previously, researchers wanting to implement sophisticated leaf energy budget models that required numerical solutions had to write their model and learn a numerical algorithm to solve it. Most often, these solutions are not published and/or are not open source. This slows down research for non-specialists by introducing unnecessary barriers and can be error-prone. For example, the current **tealeaves** model relies on previous work by Foster and Smith (1986). Without a platform like **tealeaves**, extending their work required developing the mathematical and computational tools *de novo* every time. Also, the published version of Foster and Smith (1986) contains several small errors and typographical inconsistencies in the equations. While these are most likely mistakes made during typesetting and publication, without open source code, it is very challenging to determine if these mistakes also occurred in their computer simulations. Transparent, open source code does not prevent mistakes, but makes it easier for the community to discover mistakes and fix them faster.

The **tealeaves** model is more complex than most other leaf energy balance models in the literature, which makes it more flexible and realistic but more computationally intensive. For example, the R package **plantecophys** (Duursma 2015) uses the isothermal net radiation approximation of Leuning *et al.* (1995). This reduces the number of iterations and speeds up computation by assuming that long-wave radiative flux from the leaf is proportional to air rather than leaf temperature. Other recent models (e.g. Buckley *et al.* 2014; Schymanski and Or 2017) assume free convection or do not account for virtual temperature differences in moist air (Equation 14) (Okajima *et al.* 2012; Duursma 2015). Other models, except Okajima *et al.* 2012, also do not account for different surrounding temperatures above and below the leaf surface ($T_{sky}$ and $T_{air}$, respectively) when calculating thermal infrared radiation (see Equation 3). These simplifications may be adequate for many applications and the benefit in speed may be necessary for scaling up. However, recent comparisons of simple and complex energy balance models in the field found that the complex models, like that implemented in **tealeaves**, fit the observed distribution of wheat leaf canopy temperatures better (Webber *et al.* 2017). Future development of **tealeaves** will make it easier to implement simpler models. A full range of models, from simple to complex, will serve the broadest range of applications and allow users to test when simplifying assumptions are justified. To facilitate this, future versions of **tealeaves** will allow users to provide functions rather than values for some parameters. For example, a less computationally intensive model could be contructed by using a function for calculating boundary layer conductances that assume free convection is negligible. Or users could substitute a more appropriate function for their application, such as cloudy rather than clear skies in the case of Equation 3.

Ultimately, the goal of **tealeaves** is to provide a platform for implementing realistic and fully customizable energy budget models. Such models may take too much computational time to be useful for large-scale ecosystem models, but they can help understand a wider range of fascinating and poorly understood leaf anatomical and morphological features. The Introduction lists several possible uses, but most of these problems cannot currently be solved with **tealeaves** alone. For example, many photosynthetic processes are temperature-sensitive, but it would require simultaneous modelling of leaf temperature, stomatal conductance and photosynthesis to predict optimal trait values. **tealeaves** intentionally does not specify these other models because the concept is that it should stand alone and be able to interact with many other models. For example, **tealeaves** could be combined with a stomatal response function such as the Ball–Berry model (Ball *et al.* 1987) or found through optimization (Buckley *et al.* 2014; Duursma 2015; Muir 2019). Similarly, the leaf temperature throughout a canopy could be modeled by running **tealeaves** over a gradient of light, wind, temperature and so forth. **tealeaves** should therefore be thought of as one component in an expanding ecosystem of interrelated tools for modelling plant physiology.

Currently, **tealeaves** has several limitations that I plan to address in future releases. It uses rather simple models of infrared radiation and direct versus diffuse radiation. Ideally, it would be better if users could supply their own functions to calculate these parameters from the total irradiance. The model also assumes leaves are horizontal, whereas leaf orientation varies widely. Following previous authors, I modeled heat transfer as a mixed convection (Equations 9 and 27), but this may not adequately describe real leaf heat exchange (Roth-Nebelsick 2001). **tealeaves** calculates equilibrium as opposed to transient behavior (Vialet-Chabrand and Lawson 2019), which may takes several minutes to reach. Finally, the model assumes a single homogenous leaf temperature rather than using finite element modelling to calculate leaf temperature gradients across leaves of different shapes. These are important limitations of the current software which can be addressed in future work.

In conclusion, **tealeaves** provides new software for leaf energy balance models in R. Leaf energy balance models are highly useful tools for understanding plant form and function and new computational tools will make these models more broadly accessible, advancing basic and applied plant science.

## Data

Annotated source code to generate this manuscript is available on GitHub (https://github.com/cdmuir/tealeaves-ms) and archived on Zenodo (https://doi.org/10.5281/zenodo.167281492). A development version of **tealeaves** is currently available on GitHub (https://github.com/cdmuir/tealeaves) and the version used for this manuscript is archived on Zenodo (https://doi.org/10.5281/zenodo.2808079).

## Supporting Information

The following additional information is available in the online version of this article—

**Table S1.** Reasonable values for **tealeaves** parameter inputs with references to the primary literature. The current version of **tealeaves** uses a default value within the range of reasonable values. See Table 1 for a key to symbols.

**Figure S1.** The effect of key leaf (A–C) and environmental (D–F) parameters on leaf temperature, holding other parameters constant. (A) Greater stomatal conductance ($g_{sw}$ , *x*-axis) reduces leaf temperature through latent heat loss. (B) Larger leaves (*d*, *x*-axis) have thicker boundary layers, causing them to heat up more in the sun. (C) Amphistomatous leaves (SR = 0.5, *x*-axis) lose more water through transpiration than leaves with all stomata on one surface, leading to a lower leaf temperature. (D) Greater humidity (RH, *x*-axis) increases leaf temperature by limiting latent heat loss. (E) With low solar radiation ($S_{sw}$, *x*-axis), leaf temperature is below air temperature; with high solar radiation, leaf temperature is greater than air temperature. (F) At greater wind speeds (*u*, *x*-axis), leaf temperature is more closely coupled to air temperature. The discontinuity represents the shift from laminar to turbulent flow. For reference, the dashed line is the air temperature in all simulations. All calculations used the following leaf parameter values unless they varied: *d* = 0.1 m; $\alpha_{s}=0.5$; $\alpha_{l}=0.97$; $g_{sw}=5\;\mu molm^{-2}s^{-1}Pa^{-1}$; $g_{uw}=0.1\;\mu molm^{-2}s^{-1}Pa^{-1}$; SR=0.5. All calculations used the following environmental parameter values unless they varied: *P* = 101.3246 kPa; *r* = 0.2; RH=0.5; $S_{sw}=1000$ W m^−2^; $T_{air}=298.15$ K; *u* = 2 m s^−1^. See Table 1 for symbol definitions.

plz054_suppl_Supplementary_Material

## Sources of Funding

This study received startup funds from the University of Hawai’i.

## Conflict of Interest

None declared.

## Appendix: Theory

This appendix describes how the components of leaf energy budgets (absorbed radiation, thermal infrared radiation loss, sensible heat flux and latent heat flux) are modelled in **tealeaves**. The model largely follows that previously developed by Foster and Smith (1986), so for brevity I have not explained every equation. I refer interested readers to Nobel (2009), Monteith and Unsworth (2013) and Jones (2014) for more background on the biophysics.

### Absorbed radiation

The **tealeaves** model for absorbed radiation follows Okajima *et al*. (2012):

$$\begin{array}{l} R_{abs}=\alpha_{s}(1+r)S_{sw}+\alpha_{l}\sigma \left(T_{sky}^{4}+T_{air}^{4}\right). \end{array}$$ (2)

The left half of the equation calculates absorbed solar radiation. $\alpha_{s}$ is the fraction of solar radiation absorbed. $S_{sw}$ is the total incident radiation. This is multiplied by 1 + *r*, where *r* is the albedo of soil, to account for direct and reflected sunlight. The right half includes thermal infrared radiation. $\alpha_{l}$ is the fraction of thermal infrared radiation absorbed. *σ* is the Stefan–Boltzmann constant. As in Okajima *et al*. (2012), the clear sky temperature ($T_{sky}$) is calculated as a function of air temperature ($T_{air}$):

$$\begin{array}{l} T_{sky}=T_{air}-\frac{20S_{sw}}{1000}. \end{array}$$ (3)

Okajima *et al*. (2012) define $T_{sky}$ as the ‘effective sky radiation temperature’ of ‘cloudless sky’.

### Thermal infrared radiation loss

Both leaf surfaces lose thermal infrared radiation as a function of leaf emissivity (equal to the infrared absorption, $\alpha_{l}$) and air temperature (Foster and Smith 1986; Okajima *et al*. 2012):

$$\begin{array}{l} S_{r}=2\sigma \alpha_{l}T_{leaf}^{4}. \end{array}$$ (4)

### Sensible heat flux

Sensible heat flux (*H*) is calculated as:

$$\begin{array}{l} H=P_{a}c_{p}g_{h}(T_{leaf}-T_{air}). \end{array}$$ (5)

*c*_*p*_ is the heat capacity of air. The density of dry air ($P_{a}$) is calculated as in Foster and Smith (1986):

$$\begin{array}{l} P_{a}=\frac{2P}{R_{air}(T_{leaf}-T_{air})}. \end{array}$$ (6)

*P* is the atmospheric pressure and $R_{air}$ is the specific gas constant for dry air. **tealeaves** sums the boundary layer conductance to heat for both the upper and lower surface following Foster and Smith (1986), assuming a horizontal leaf orientation:

$$\begin{array}{l} g_{h}=\frac{D_{h}Nu}{d}. \end{array}$$ (7)

Nu is the unitless Nusselt number (defined below) and *d* is the characteristic leaf dimension, a thermally relevant measure of leaf size. The diffusion coefficient of heat in air (*D*_h_) is a function of temperature and pressure:

$$\begin{array}{l} D_{h}=D_{h,0}{\left(\frac{T}{273.15}\right)}^{eT}\frac{101.3246}{P}. \end{array}$$ (8)

The temperature dependence of diffusion (eT) is generally between 1.5 and 2 for heat and water vapour (Monteith and Unsworth 2013). To calculate diffusion coefficients, **tealeaves** uses the average of the leaf and air temperature: $T=(T_{air}+T_{leaf})/2$. The Nusselt number Nu is modeled as a mixed convection:

$$\begin{array}{l} Nu^{3.5}={Nu}_{forced}^{3.5}+{Nu}_{free}^{3.5} \end{array}$$ (9)

where

$$\begin{array}{l} Nu_{forced}=aRe^{b} \end{array}$$ (10)

$$\begin{array}{l} Nu_{free}=cGr^{d}. \end{array}$$ (11)

a,b,c,d are constants that depend on whether flow is laminar or turbulent and the direction of flow in the case of free convection (see below). In general, when the Archimedes number (also called the Richardson number) $Ar=Gr/Re^{2}\ll 0.1$, free convection dominates; when $Ar=Gr/Re^{2}\gg 10$, forced convection dominates (Nobel 2009). The Nusselt number coefficients can be found in Monteith and Unsworth (2013). For forced convection, flow is laminar if Re<4000, a=0.6,b=0.5; flow is turbulent if Re>4000, a=0.032,b=0.8. These cut-offs for leaves are lower than for artificial surfaces because trichomes and other anatomical features of leaf surfaces induce turbulence more readily (Grace and Wilson 1976). For free convection, flow is laminar. For the upper surface when $T_{leaf}>T_{air}$ or the lower surface when $T_{leaf}<T_{air}$, c=0.5,d=0.25 . Conversely, for the lower surface when $T_{leaf}>T_{air}$ or the upper surface when $T_{leaf}<T_{air}$, c=0.23,d=0.25.

Grashof and Reynolds numbers approximate the ratio of bouyant or inertial (numerator in equations below), respectively, to viscous forces (denominator in equations below). They are calculated as:

$$\begin{array}{l} Gr=\frac{Gd^{3}|T_{v,leaf}-T_{v,air}|}{T_{air}D_{m}^{2}} \end{array}$$ (12)

$$\begin{array}{l} Re=\frac{ud}{D_{m}}. \end{array}$$ (13)

*G* is the gravitational constant and *u* is wind speed. The diffusion coefficient for momentum in air (*D*_m_) is calculated for a given temperature following the same procedure above for heat diffusion (*D*_h_; see Equation 8). The virtual temperature is calculated according to Monteith and Unsworth (2013) assuming that the leaf airspace is fully saturated while the air is has a vapour-pressure deficit of $p_{air}$:

$$\begin{array}{l} T_{v,air}=T_{air}/(1-(1-\varepsilon )(p_{air}/P)) \end{array}$$ (14)

$$\begin{array}{l} T_{v,leaf}=T_{leaf}/(1-(1-\varepsilon )(p_{sat}/P)). \end{array}$$ (15)

ε is the ratio of water to air molar masses. The saturation water vapour pressure $p_{sat}$ as a function of temperature is calculated using the Goff–Gratch equation (Vömel 2016). The vapour pressure of air is calculated from the relative humidity (RH) as $p_{air}=RHp_{sat}$.

### Latent heat flux and evaporation

Latent heat flux is the product of the latent heat of vaporization, the total leaf conductance to water vapour and the water vapour gradient:

$$\begin{array}{l} L=h_{vap}g_{tw}d_{wv}. \end{array}$$ (16)

The latent heat of vapourization ($h_{vap}$) is a linear function of temperature. **tealeaves** calculates $h_{vap}$ using parameters estimated from linear regression on data from Nobel (2009):

$$\begin{array}{l} h_{vap}=56847.68250\;[J\;mol^{-1}]-43.12514\;[J\;mol^{-1}\;K^{-1}]T[K]. \end{array}$$ (17)

The water vapour pressure differential ($d_{wv}$) from the inside to the outside of the leaf is the water vapor pressure inside the leaf, which is assumed to be saturated ($p_{leaf}=p_{sat}$), minus the water vapor pressure of the air ($p_{air}$), calculated as described above. This value is converted from kPa to mol m^−3^ using the ideal gas law:

$$\begin{array}{l} d_{wv}=p_{leaf}/(\bar{R}T_{leaf})-RHp_{air}/(\bar{R}T_{air}). \end{array}$$ (18)

$\bar{R}$ is the ideal gas constant. The total conductance to water vapor ($g_{tw}$) is the sum of the parallel lower (usually abaxial) and upper (usually adaxial) conductances

$$\begin{array}{l} g_{tw}=g_{w,lower}+g_{w,upper}. \end{array}$$ (19)

The conductance to water vapor on each surface is a function of parallel stomatal ($g_{sw}$) and cuticular ($g_{uw}$) conductances in series with the boundary layer conductance ($g_{bw}$). The stomatal, cuticular and boundary layer conductance on the lower surface are:

$$\begin{array}{l} g_{sw,lower}=[g_{sw}(1-SR)][R(T_{leaf}+T_{air})/2] \end{array}$$ (20)

$$\begin{array}{l} g_{uw,lower}=(g_{uw}/2)[R(T_{leaf}+T_{air})/2]. \end{array}$$ (21)

Note that the user provides the total leaf stomatal and cuticular conductance to water vapur in units of µmol m^−2^ s^−1^ Pa^−1^, which are then converted to units of m s^−1^ using the ideal gas law. Stomatal conductance is partitioned among leaf surfaces depending on stomatal ratio (SR); cuticular conductance is assumed equal on each leaf surface. The corresponding expressions for the upper surface are:

$$\begin{array}{l} g_{sw,upper}=(g_{sw}SR)[R(T_{leaf}+T_{air})/2] \end{array}$$ (22)

$$\begin{array}{l} g_{uw,upper}=g_{uw,lower}. \end{array}$$ (23)

Partitioning total stomatal and cuticular conductance to water vapor across surfaces is not typically part of leaf energy budget models. However, stomatal (Muir 2015) and cuticular (Karbulková *et al*. 2008) conductance can differ dramatically on each surface, so it could be useful modify these parameters, especially if leaf temperature models are integrated with photosynthetic models (Muir 2019).

The boundary layer conductances for each surface differ because of free convection (Foster and Smith 1986) and are calculated similarly to that for heat (Equation 7):

$$\begin{array}{l} g_{bw}=\frac{D_{w}Sh}{d}. \end{array}$$ (24)

The diffusion coefficient for water vapor in air at a given temperature (*D*_w_) is calculated using the Equation 8, except that is *D*_w,0_ is substituted for *D*_h,0_. Each surface has its own Sherwood number (Sh):

$$\begin{array}{l} {\text{Sh}}_{\text{forced}}={\text{Nu}}_{\text{forced}}{\left(D_{h}/D_{w}\right)}^{\frac{1}{3}} \end{array}$$ (25)

$$\begin{array}{l} {\text{Sh}}_{\text{free}}={\text{Nu}}_{\text{free}}{\left(D_{h}/D_{w}\right)}^{\frac{1}{4}}. \end{array}$$ (26)

As with Nu, Sh is calculated assuming mixed convection:

$$\begin{array}{l} Sh^{3.5}={Sh}_{forced}^{3.5}+{Sh}_{free}^{3.5}. \end{array}$$ (27)

Evaporation rate (mol H_2_O m^−2^ s^−1^) is the product of the total conductance to water vapour (Equation 19) and the water vapour gradient (Equation 18):

$$\begin{array}{l} E=g_{tw}d_{wv}. \end{array}$$ (28)
