# Supplementary material for: tealeaves: an R package for modelling leaf temperature using energy budgets
Source: AoB Plants. 2019 Dec 8;11(6):plz054. doi: 10.1093/aobpla/plz054 (PMC6899345; doi:10.1093/aobpla/plz054)
Supplement: plz054_suppl_Supplementary_Material [file plz054_suppl_supplementary_material.pdf]

Supporting Information

Supporting Tables

Table S1: Reasonable values for **tealeaves** parameter inputs with references to the primary literature. The current version of **tealeaves** uses a default value within the range of reasonable values. See Table 1 for a key to symbols.

Symbol	**tealeaves** Default	Range	Reference(s)
**Leaf parameters:**			
d	0.1	0.004 – 0.4 m	Wright *et al.* (2017)
α_l	0.97	0.95 – 0.97	Gutschick (2016)
α_s	0.5	0.4 – 0.6	Jones (2014)
g_{sw}	5	0.01 – 10 μmol m^{-2} s^{-1} Pa^{-1}	Lin *et al.* (2015); Duursma *et al.* (2019)
g_{uw}	0.1	0.01 – 1 μmol m^{-2} s^{-1} Pa^{-1}	Duursma *et al.* (2019)
SR	0.5	0 – 1 (untransformed)	Muir (2015)
**Environmental parameters:**			
P	101.3246	50 (5000 mas†) – 100 (0 mas) kPa	Körner (2007); Stull (2011)
r	0.2	0.1 (lava) – 0.6 (ice)	Stull (2011)
RH	0.5	0 – 1	Jones (2014)
S_{sw}	1000	0 – 1000 W m^{-2}	Jones (2014)
T_{air}	298.15	270 – 320 K	Jones (2014)
u	2	0 – 10 m s^{-1}	Vogel (2009)

† meters above sea level

Supporting Figures

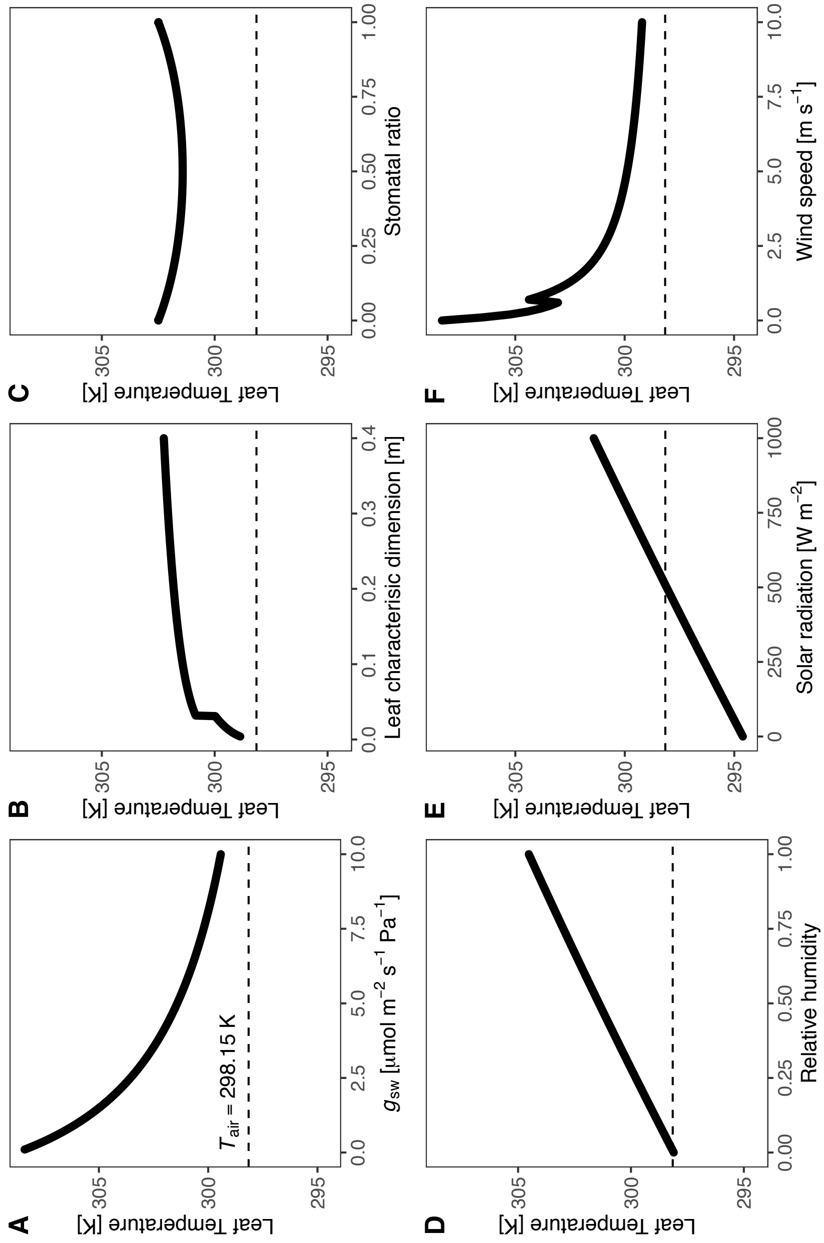
A
B
C
D
E
F
Leaf Temperature [K]
305
300
295
T_{air} = 298.15 K
0.0 2.5 5.0 7.5 10.0
g_{sw} [µmol m^{-2} s^{-1} Pa^{-1}]
0.0 0.1 0.2 0.3 0.4
Leaf characterisic dimension [m]
0.00 0.25 0.50 0.75 1.00
Stomatal ratio
Relative humidity
0 250 500 750 1000
Solar radiation [W m^{-2}]
Wind speed [m s^{-1}]

Figure S1: The effect of key leaf (**A** − **C**) and environmental (**D** − **F**) parameters on leaf temperature, holding other parameters constant. **A**) Greater stomatal conductance (g_{sw}, x-axis) reduces leaf temperature through latent heat loss. **B**) Larger leaves (d, x-axis) have thicker boundary layers, causing them to heat up more in the sun. **C**) Amphistomatous leaves (SR = 0.5, x-axis) lose more water through transpiration than leaves with all stomata on one surface, leading to a lower leaf temperature. **D**) Greater humidity (RH, x-axis) increases leaf temperature by limiting latent heat loss. **E**) With low solar radiation (S_{sw}, x-axis), leaf temperature is below air temperature; with high solar radiation, leaf temperature is greater than air temperature. **F**) At greater wind speeds (u, x-axis) leaf temperature is more closely coupled to air temperature. The discontinuity represents the shift from laminar to turbulent flow. For reference, the dashed line is the air temperature in all simulations. All calculations used the following leaf parameter values unless they varied: $d = 0.1$ m; $\alpha_{\mathrm{s}} = 0.5$; $\alpha_{\mathrm{l}} = 0.97$; $g_{\mathrm{sw}} = 5$ μmol m^{-2} s^{-1} Pa^{-1}; $g_{\mathrm{uw}} = 0.1$ μmol m^{-2} s^{-1} Pa^{-1}; SR = 0.5. All calculations used the following environmental parameter values unless they varied: $P = 101.3246$ kPa; $r = 0.2$; RH = 0.5; $S_{\mathrm{sw}} = 1000$ W m^{-2}; $T_{\mathrm{air}} = 298.15$ K; $u = 2$ m s^{-1}. See Table 1 for symbol definitions.

Extended examples

R code for running extended examples (Fig. 2). The below code and the code to generate figures are deposited on GitHub (https://github.com/cdmuir/tealeaves-ms).

```
# Extended example 1:
# leaf size and leaf-to-air temperature differential

library(tealeaves)
lp  <- make_leafpar(
  replace = list(
    leafsize = set_units(c(0.005, 0.1, 0.4), "m")
  )
)
ep <- make_enviropar(
  replace = list(
    S_sw = set_units(660, "W/m^2"),
    T_air = set_units(seq(278.15, 308.15, 5), "K")
  )
)
exe1 <- tleaves(lp, ep, cs, progress = TRUE, quiet = TRUE,
                set_units = TRUE, parallel = TRUE)
```

```
# Extended example 2:
# Solar radiation and leaf-to-air temperature differential

library(tealeaves)
lp  <- make_leafpar(
  replace = list(
    g_sw = set_units(c(1, 3, 5), "umol/m^2/s/Pa")
  )
)
ep <- make_enviropar(
  replace = list(
    S_sw = set_units(seq(50, 950, 100), "W/m^2")
  )
)
exe2 <- tleaves(lp, ep, cs, progress = TRUE, quiet = TRUE,
                set_units = TRUE, parallel = TRUE)
```

```
# Extended example 3:
# wind speed and leaf-to-air temperature differential

library(tealeaves)
lp  <- make_leafpar(
  replace = list(
    leafsize = set_units(c(0.005, 0.1, 0.5), "m")
  )
)
ep <- make_enviropar(
  replace = list(
    wind = set_units(exp(seq(log(0.01), log(10),
                             length.out = 1e2)), "m/s")
  )
)
exe3 <- tleaves(lp, ep, cs, progress = TRUE, quiet = TRUE,
                set_units = TRUE, parallel = TRUE)
```

```
# Extended example 4:
# Stomatal ratio and evaporation

library(tealeaves)
lp  <- make_leafpar(
  replace = list(
    g_sw = set_units(c(0.4, 4), "umol/s/m^2/Pa"),
    logit_sr = set_units(seq(-10, 10, length.out = 1e2))
  )
)
ep <- make_enviropar(
  replace = list(
    RH = set_units(0.2),
    T_air = set_units(293.15, "K"),
    wind = set_units(c(0, 2), "m/s")
  )
)
exe4 <- tleaves(lp, ep, cs, progress = TRUE, quiet = TRUE,
                set_units = TRUE, parallel = TRUE)
```
